# Genomic Fingerprint Associated with Familial Idiopathic Pulmonary Fibrosis: A Review

**DOI:** 10.7150/ijms.80358

**Published:** 2023-01-31

**Authors:** Dongyan Ding, Rong Gao, Qianfei Xue, Rumei Luan, Junling Yang

**Affiliations:** 1 Department of Respiratory Medicine, The Second Hospital of Jilin University, Changchun, China.; 2 Hospital of Jilin University, Changchun, China.

**Keywords:** Familial idiopathic pulmonary fibrosis, Sporadic idiopathic pulmonary fibrosis, Telomerase-associated gene, Mucin 5B, Surfactant-related gene.

## Abstract

Idiopathic pulmonary fibrosis (IPF) is a severe interstitial lung disease; although the recent introduction of two anti-fibrosis drugs, pirfenidone and Nidanib, have resulted in a significant reduction in lung function decline, IPF is still not curable. Approximately 2-20% of patients with IPF have a family history of the disease, which is considered the strongest risk factor for idiopathic interstitial pneumonia. However, the genetic predispositions of familial IPF (f-IPF), a particular type of IPF, remain largely unknown. Genetics affect the susceptibility and progression of f-IPF. Genomic markers are increasingly being recognized for their contribution to disease prognosis and drug therapy outcomes. Existing data suggest that genomics may help identify individuals at risk for f-IPF, accurately classify patients, elucidate key pathways involved in disease pathogenesis, and ultimately develop more effective targeted therapies. Since several genetic variants associated with the disease have been found in f-IPF, this review systematically summarizes the latest progress in the gene spectrum of the f-IPF population and the underlying mechanisms of f-IPF. The genetic susceptibility variation related to the disease phenotype is also illustrated. This review aims to improve the understanding of the IPF pathogenesis and facilitate his early detection.

## Introduction

Idiopathic pulmonary fibrosis (IPF) is a form of idiopathic interstitial pneumonia (IIP) characterized by progressive lung scarring, persistent decline in lung function, and poor prognosis 1. Among all IIPs, IPF is the most common and fatal type with the worst prognosis. Approximately 80% of all patients with familial IIP are diagnosed with IPF 2. Patients with IPF have a shorter disease course than those with other types of IIP. The median survival of patients with IPF after diagnosis is only 2-4 years 3, and the clinical course and survival rates vary greatly 4. Pirfenidone and Nidanib are anti-fibrotic drugs approved by the US Food and Drug Administration 5, which can delay, but not prevent or improve, the decline of lung function. To date, lung transplantation is the only known life-prolonging intervention 6. The etiology of PF has not been fully established; however, it is thought to be caused by environmental factors and genetic susceptibility 7.

IPF occurs in sporadic, familial, or syndromic forms 8. The familial form accounts for 20% of all IPF cases, hereafter referred to as f-IPF 9. However, 80% of occurrences of IPF (the great majority) have not been divided among the two other forms. One of them could be rare, or they could be equally common. Sporadic IPF (s-IPF) is multifactorial; in addition to environmental risk factors, it is occasionally associated with polymorphic variants of various genes. Risk factors include smoking, inhaling metal and wood dust particles, and exposure to particular medications, such as methotrexate 2. Moreover, males have been identified to be more at risk than females. The clinical symptoms of f-IPF and s-IPF are similar; however, f-IPF is caused by gene variation and characterized by the earlier onset of symptoms 8. An increasing number of reports on PF in familial clusters and some rare genetic diseases suggest that genetic factors play an important role in IPF 7. The incidence of IPF and rate of disease progression differ in patients with the same levels of exposure, indicating the influence of genetic background heterogeneity.

Furthermore, IPF may occur in a familial setting, and patients with IPF show familial aggregation in different environments, suggesting a genetic cause 10. IPF has been reported in monozygotic twins raised in different environments from an early age 11. Additionally, IPF has been detected in successive generations within families 12 and family members separated at an early age 13. The known familial incidence of IPF provides evidence of the genetic background of the disease 14. Genetic testing may reveal the necessity of screening for other etiopathogenesis factors of IPF 15, including multisystem genetic disorders such as dyskeratosis congenita (DC) 16, neuroendocrine cell hyperplasia of infancy 17, Niemann*-*Pick disease 18, Hermansky-Pudlak syndrome, and short telomere syndrome 14.

Family cases of IPF were first described in 1907 by Sandoz 19 in Germany, who described a pair of 18-year-old twin sisters who both died of slowly progressive respiratory failure and whose autopsies revealed terminal honeycomb. Between 2% and 20% of patients with IPF have a first-degree relative with DPLD 20. Since the earliest description of IPF, familial clusters of similar cases have been identified as familial PF or f-IPF, which is defined by at least two biological family members with clinical and histological features of IPF 21. The occurrence of familial cases of PF raises the possibility of a genetic basis, and the search for the underlying genetic determinants of this disease through familial aggregates can better elucidate the pathogenesis of familial and more common sporadic diseases.

However, it must first be speculated whether the sporadic and familial forms of IPF are indeed distinct manifestations of a single clinical entity, or whether they represent two distinct disease processes. Among relatives with f-IPF, the most common phenotype of interstitial lung disease (ILD) is usual interstitial pneumonia (UIP); however, in at least 50% of known familial cases, affected family members develop more than one ILD phenotype 14. The Mayo Clinic series further concluded that f-IPF and s-IPF share the same clinical, radiological, and pathological features 12, except that the average age of onset of f-IPF (55 years) is lower than that of s-IPF (68 years). In addition, the average age of diagnosis for f-IPF (58 years) is lower than that for s-IPF (65 years) 22.

Genetic factors play an important role in both s-IPF and f-IPF 23, especially in patients with f-IPF. This finding suggests that a single autosomal dominant mechanism of reduced penetration cannot exclude autosomal recessive inheritance or heterogeneous coexistence of genetic traits 24; however, the genetic pattern of f-IPF remains unclear. Current data suggest that at least one-third of the risks of s-IPF or f-IPF can be attributed to common genetic variants 25, some of which have prognostic significance for patients with IPF 26. In addition, more rare genetic variants that affect susceptibility to f-IPF have been identified 23.

Susceptibility gene variation is informative for clarifying the pathogenesis of f-IPF and determining its key disease-causing targets. To date, there are no clinical guidelines on when and which gene tests should be performed in patients with IPF. Previous research has examined genetic associations, common single nucleotide polymorphisms (SNP) and rare gene mutations are associated with their development 25. Rare mutations in genes telomerase reverse transcriptase (*TERT*) 27, telomerase RNA component (*TERC*) 28, dyskeratosis congenita 1 (*DKC1*) 29, telomere repeat binding factor 1-interacting nuclear factor 2 (*TINF2*) 30, regulator of telomere length 1 (*RTEL1*), zinc finger CCHC-type containing 8 (*ZCCHC8*), poly(A)-specific ribonuclease (*PARN*) 31, surfactant protein C (*SFTPC*) 32, surfactant protein A2 (*SFTPA2*) 33, the ATP-binding cassette-type family A member 3 transporter (*ABCA3*) 34), and common variants of 10 loci (3q26, 4q22, 5p15, 6p24, 7q22, 10q24, 11p15, 13q34, 15q14-15, and 19p13) 25 are associated with f-IPF.

### Telomere-associated genes

**Fig 1:**
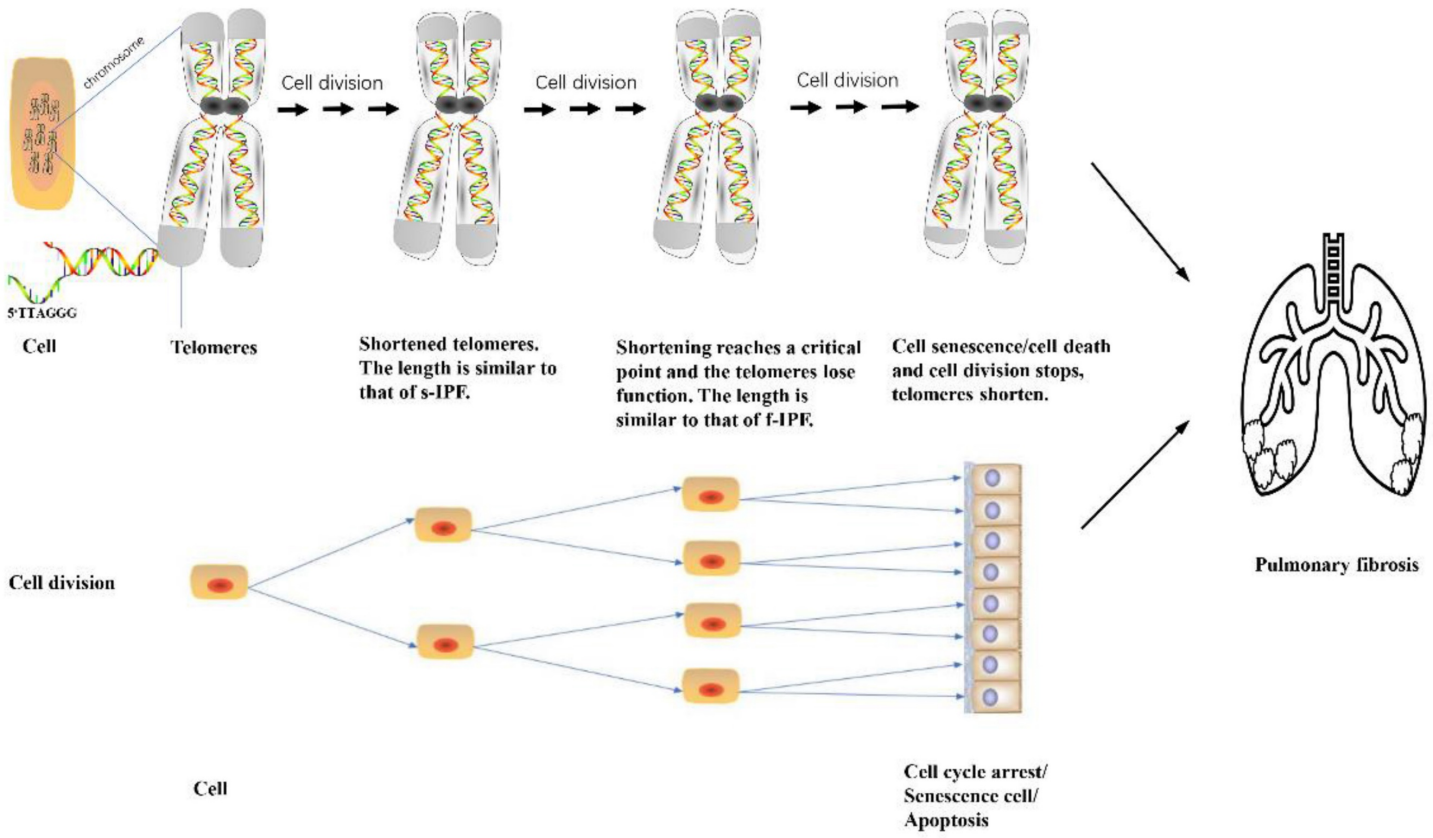
Role of short telomeres in PF.

Telomeres are cap-like structures at the ends of chromosomes comprising multiple repeated 5ʹ-TTAGGG-3ʹ DNA sequences that protect chromosome integrity and stability during cell division 35. As the cells divide, the telomere length progressively shrinks 36. Patients with familial and, less commonly, sporadic IPF have extremely short telomeres. TERC and TERT are the two main components that constitute the telomerase complex 37, a specialized ribonucleic protein and major component of the mechanism that maintains telomere lengthening 38. Telomerases are found in germlines, stem cells, tissue cells, and most cancer cells, with the ability to proliferate actively, but are scarce or absent in normal somatic cells 39. Mutations in genes that regulate telomere lengthening can negatively affect their maintenance, which can lead to gradual telomere shortening. When telomeres are shortened to a certain critical point, DNA damage reaction is activated, mitochondria cannot divide, and apoptosis ensues 40 (Fig. 1). Telomere shortening is related to aging and senile diseases 41, as telomere length decreases with age 36. Telomere mutations are found in 25% of patients with f-IPF and 1-3% of those with s-IPF 42.

Telomere shortening occurs gradually throughout the life cycle as cells undergo continuous mitosis, especially in individuals genetically susceptible to familial IPF who have significantly shorter telomeres than patients with sporadic IPF. The mutation of the telomerase complex accelerates telomere shortening. When telomere shortening reaches a critical value, DNA damage is activated, and mitochondria fail to divide, resulting in cell senescence, apoptosis, or cell division cycle termination.

**Fig 2:**
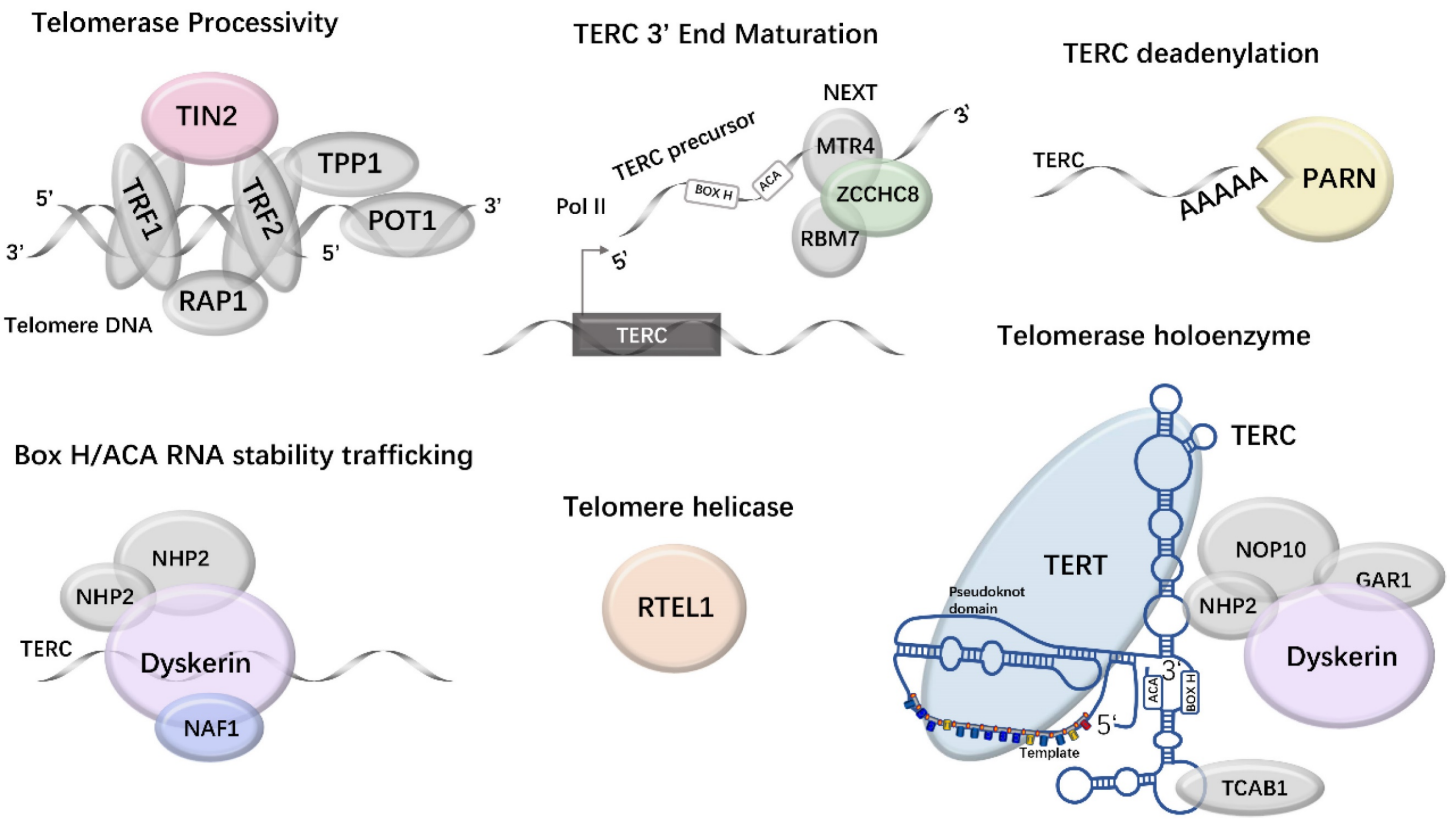
** Eight telomerase and telomere maintenance genes are mutated in f-IPF.** The mutated components are shown in color.

Half of IPF families with known mutations carry TERT or TERC mutations 43. Half member might carry mutations in one or more of the other telomere maintenance genes, such as *RTEL1*, *PARN*, nuclear assembly factor 1 (*NAF1*), *TINF2*, *ZCCHC8*, and *DKC1*(Fig. 2). Four genes that affect *TERC* mRNA deadenylation, TERC trafficking, and stability are *PARN*, *NAF1*, *ZCCHC8*, and *DKC1*. Fig. 2 shows how they indirectly affect telomere maintenance.

#### Telomerase

Telomerase inactivation is due to the silence of TERT expression 44. TERT mutations are associated with short telomere syndrome and a wide range of clinical phenotypes, such as congenital dyskeratosis, bone marrow failure, liver disease, and PF 45. Age-related PF is usually the first indicator of genetic defects in families with TERT mutations 46, with an age at onset of 60-70 years 47, and mutations in telomerase genes are the most common inherent risk factors for f-IPF 48. Families with telomerase gene mutations for f-IPF show an expected genetic predisposition 49. Germline *TERT* mutations lead to haploinsufficiency, which in turn leads to telomere shortening 50. These shorter telomeres are inherited over generations. This is why TERT mutations are considered pathogenic; however, it may take over 300 years before the cumulative effect of hereditary telomere shortening leads to PF 51. Ultimately, in these families, the onset of the disease is anticipated. This is followed by successive generations with increased symptoms of an earlier onset 52. This finding explains the clinical complexity of *TERT* mutations, which are based on randomly occurring mutations and hereditary genetic defects.

The non-coding RNA component of telomerase *TERC* provides a structural scaffold for the formation and action of telomerase complexes 53. It is used for the synthesis of template regions and progressive telomere repeats 54. *TERC* RNA is made up of 451 nucleotides, but longer transcripts can be identified by reverse transcription-polymerase chain reaction 55 and cDNA 3' end 56.

Diaz de Leon et al. assessed 134 patients with f-IPF from 21 families with PF (all with telomerase *TERT* mutations) and found that 18% of the patients had heterozygous *TERT* mutations, covering 18 mutation sites, and some patients may have several telomerase mutations 57. Telomerase gene mutations have been reported in approximately 10% of patients with s-IPF, and in approximately 25% of patients with f-IPF 48. Armanios et al. observed 73 families with f-IPF, six of whom (8%) had shorter telomeres in peripheral blood leukocytes than those observed in their peers, and *TERT/TERC* heterozygous mutations encoding RNA ligands of the telomerase complex in an autosomal dominant inheritance pattern 43. *TERT* and *TERC* mutations have also been validated in another study in families with f-IPF 58.

#### RTEL1

RTEL1 is a DNA helicase that unlocks the T-ring structure of telomeres at the end of chromosomes 59. Mutations in* RTEL1* result in the cleavage of telomere ends near the T-loop by the structure-specific endonuclease subunit (SLX4), which leads to the release of telomeric repeats (T-circles). These successive DNA cycle mutations gradually generate shortened telomeres 60. *RTEL1* mutants have very short telomeres, whose length is similar to those of peripheral blood mononuclear cells (PBMCs) in individuals with *TERT*, *TERC*, and *DKC1* mutations 61. Damage to *RTEL1* also leads to genomic instability and replication defects 62, which result in disruption of cell cycle progression. Short telomeres are present in approximately one-third of patients with familial ILD, and rare variations in *RTEL1* are associated with the development of familial ILD, especially in patients with short telomeres in PBMCs 61.

#### PARN

*PARN* is a widely expressed enzyme that removes poly(A) tails by cap-dependent deadenylation of mRNAs and, in doing so, typically plays a role in regulating global mRNA levels during development 63, as well as other more specialized functions, including Dicer-independent end pruning of microRNA miR-451 64 and deadenylation of small nucleolar RNAs (snoRNAs) 65. It contributes to the poly(A)-specific maturation and telomere length maintenance in fibroblasts and induces pluripotent stem cells 66. *PARN* haploinsufficiency may lead to telomere shortening through a decrease in small nucleolar RNAs of the H/ACA box or *TERC* RNA composition. *PARN* mutations cause diseases that have been related to telomere length, including f-IPF 67 and DC 68. Carriers of *PARN* and *RTEL1* mutations have shortened leukocyte telomere lengths, and family members who did not inherit *PARN* or *RTEL1* mutations have shorter mean telomere lengths than unrelated individuals (i.e., spouses) but longer telomere lengths than carriers of* PARN* or *RTEL1* mutations, with epigenetic inheritance of short telomeres observed in family members 67. Together, these genes explain approximately 7% of f-IPF and highlight the link between PF and telomere dysfunction.

#### DKC1

The DC gene variant, X-linked gene *DKC1*, is another component of the telomerase complex and is also important for its stability and maintenance 69. In 1995, the first link between DKC1 and pulmonary fibrosis was found in 46 families recruited at Hammersmith Hospital which occurs in 19% of cases and is a congenital telomere disease characterized by skin abnormalities, bone marrow failure, and lung fibrosis 70. DNA analyses of two siblings who died of IPF revealed significantly shortened peripheral blood telomeres, undetectable alveolar epithelial telomeres, and a shared new A-to-G transition near the TERC-binding domain in *DKC1*; this domain would encode an amino acid substitution for Thr405Ala, a mutation that destabilizes TERC and impairs telomerase function 71. The X-linked *DKC1* variant represents a telomere-associated gene for the genetic cause of f-IPF.

#### TINF2

*TINF2* encodes a protein that is involved in telomere protection and maintenance 72. The shelterin complex, made up of six proteins, protects chromosome ends from DNA damage and regulates signaling and repair mechanisms as well as telomerase entry and telomere activity 73. TERF1-interacting nuclear factor 2, the protein product of *TINF2*, is a subunit of the shelterin complex. Mutations in *TINF2* lead to telomere shortening and the impaired recruitment of tripeptidyl-peptidase 1 (TPP1)-dependent telomerase to telomeres 74. Epithelial DNA damage response activation at chromosome ends in *TPP1*-null mice increases telomere fusion and fragility. This confirms the role of TPP1 in telomere protection, which is required for TERT recruitment to telomeres and telomere elongation *in vivo* 75. Jonathan et al. identified *TINF2* mutations in a family with PF by exome sequencing. They confirmed that *TINF2* mutations are a rare cause of f-IPF and explained the genetic risk in approximately 1% of the reported cases 30. However, *TINF2* mutations have also been reported in s-IPF 72.

#### ZCCHC8

ZCCHC8 is a component of the nuclear exosome targeting complex in mammalian cells 76. It drives the decay of predominantly short, unprocessed RNA with non-adenylated 3' termini 77, which is associated with short telomere disease 78. In single cells and model organisms, *ZCCHC8* loss leads to the accumulation of short-extended TERC forms while sacrificing mature TERC, possibly at an early stage, before PARN deadenylation 79. A genome-wide analysis identified *ZCCHC8* mutations in an adult with IPF who exhibited typical features of short telomere syndrome and a family history consistent with autosomal dominant pulmonary fibrosis; 13 affected family members were deceased.

Their TERC levels could not be measured to determine preclinical status; however, the proband's two children, who had abnormally short telomeres (shorter than those of the proband), had 50% lower TERC levels than healthy controls, and the remaining relatives had similar TERC levels to healthy controls. No *ZCCHC8* variants or other short telomere syndrome phenotypes were found in 42 genetically-unidentified families that had also been screened for PF. This suggests that these mutations are rare (1 out of 43.2%). Furthermore, this result is consistent with a loci heterogeneity found in short telomere syndrome 79. *ZCCHC8* is essential for TERC 3ʹ-terminal maturation and telomerase function, and a loss in heterozygosity causes f-IPF 79.

#### NAF1

*NAF1* is a non-coding RNA that encodes an essential protein for the biogenesis of H/ACA box small nucleolus RNA. NAF1 affects telomere activity by combining with other proteins (such as TERT) to form telomerase, which plays an important role in telomeric DNA synthesis 80. Mammalian NAF1 is essential, probably because of its conserved role in rRNA modification 81. In contrast to other box H/ACA, NAF1 has its own transcriptional regulation and is not protected by an intron lariat, so TERC may be particularly susceptible 82. Naf1 +/- mice have reduced H/ACA RNA levels, and their telomerase RNA levels were only half those of the control group, but snoRNA-guided rRNA pseudouridylation still occurred 83. Thus, NAF1 deficiency selectively disrupts telomere length homeostasis by reducing telomerase RNA levels and preserving rRNA pseudouridylation. Loss of *NAF1* function leads to short telomere syndrome, manifested as PF emphysema and telomere-mediated extrapulmonary disease, which is common in patients with s-IPF and f-IPF 83.

#### Short telomeres and PF

The association between telomere abnormality and PF is not fully understood 84. Notably, short telomeres can lead to apoptosis, senescence, and a combination of phenotypic DNA damage responses 85. Senescent cells have altered gene expression profiles and secrete a range of cytokines and growth factors (senescence-related secretory phenotypes) that may play a direct role in pathogenesis. It is hypothesized that the loss of regenerative potential of alveolar type II (AT2) epithelial cells after injury underlies telomere-associated PF, accompanied by excessive proliferation of airway cells exhibiting abnormal phenotypes 86. A study using mouse models, in which telomere repeat factor 1 was knocked out in alveolar and bronchiolar epithelial cells, has shown that the preferential activation of cellular senescence leads to inflammatory responses, upregulation of immune signaling pathways, increased mortality, lung remodeling, and spontaneous fibrosis compared with controls 87.

In the absence of telomerase mutations, s-IPF is often associated with telomere shortening; 5.71-68.2% of s-IPF patients present shorter blood leukocyte telomeres than age-matched controls, most of whom had no identifiable telomerase gene mutation, suggesting that the pathways involved in familial disease contribute to sporadic disease 88. The telomere length in PBMCs and alveolar epithelial cells is shorter in patients with f-IPF than in controls 88 and is significantly longer in the non-fibrotic region than in the fibrotic region 89 in patients with f-IPF after controlling for age and ethnicity. There is a significant difference in age at onset between the younger and older generations of patients with IPF 90, probably owing to shorter telomeres across generations. Short telomeres are disproportionately frequent in patients with s-IPF and are associated with worse survival 91.

Telomere length in peripheral blood is considered to be a marker of biological age 92; the survival of patients with IPF may be predicted by the genomic DNA telomere length. Regardless of the underlying diagnosis, faster disease progression has been observed in patients with ILD with fibrotic telomere gene mutations, suggesting that telomere length affects disease severity. Short telomeres are disproportionately present in patients with s-IPF and associated with worse survival in those with IPF 91. Observations in families with telomerase mutations suggest that emphysema is the first manifestation of telomere-associated lung disease in telomerase mutation carriers, either alone or in conjunction with PF presenting as mixed lung injury 93. No significant lung phenotypic abnormalities were found in mice with short telomeres 93. However, when mice with short telomeres were chronically exposed to cigarette smoke, they developed features of emphysema 93.

Like IPF, emphysema is associated with cumulative DNA damage in alveolar epithelial cells, with age and smoking as major risk factors 94. It follows that short telomeres represent a heterogeneous group of age-related lung diseases, of which IPF is the most common, that may be accompanied by some forms of emphysema. Patients with IPF have an annual decline in forced vital capacity of 130-210 mL, short telomeres caused by mutations in telomere-related genes, and forced vital capacity is reduced by an average of 300 mL per year 47. Short telomeres are associated with increased fibrosis, histological patterns, and mortality 95. These data support the notion that telomere function is involved in the development and progression of PF.

#### Short telomeres and lung transplantation

Regarding lung transplantations, lung epithelial cell renewal rates are high. This leads to accelerated telomere wear, senescent reprogramming of airway progenitor cells, and ultimately, airway remodeling, and fibrosis 96. In a lung transplant study of 20 patients with fibrotic ILD, eight patients presented f-IPF, and telomere shortening was found in five of them. Moreover, four patients with f-IPF had rare mutations in the telomere-related T cell receptor gamma locus (*TRG*) gene (potential risk of pathogenicity: *TERT*, *TERC*, *DCK1*, *PARN*, *RTEL1*, and *TINF2*), suggesting that telomere dysfunction is associated with adverse outcomes after transplantation. Despite that, the 1-year survival rate after transplantation was above 80%, and the procedure was beneficial to the survival of patients with fibrotic ILD, regardless of telomere dysfunction 97. Conversely, Swaminathan et al. reported a significantly increased risk of death after lung transplantation in patients carrying *TERT*, *RTEL1*, or *PARN* variants 98.

Telomere length can be inherited independently of rare genetic variants, and inheriting short telomeres (and disease risk) without inheriting the mutation is possible; this is called “occult hereditary disease” 57. Among the 75 asymptomatic first-degree relatives of patients with f-IPF, telomere length was shorter than in healthy controls, with 36% having telomere lengths less than the 10th percentile of age 99. One study showed that 97% of patients with IPF have shorter telomeres than age- and sex-matched controls 100, suggesting that short telomeres are not only found in patients with telomerase-related gene mutations associated with f-IPF. PF may be one of the few clinical symptoms of some inherited multisystem diseases, and f-IPF is the only manifestation of the disease in some DC families, members of which lack typical skin and mucosal characteristics 43.

A family history of short telomeres may be associated with neonatal respiratory distress, childhood ILD, aplastic anemia, and cryptogenic cirrhosis 101. Short telomeres and reduced keratinoid-associated undesirable protein expression are found in families with f-IPF 102; however, no mutations in *TERT*, *TERC*, *DKC1*, or other genes known to induce DC were detected 103, suggesting that the abnormal regulation of the telomerase complex is another mechanism of telomere shortening in patients with PF. However, the proportion of telomerase mutations in f-IPF was not high, indicating that other factors contributing to telomere shortening should be studied.

### Mucin 5B genes

In normal lungs, the airway secretion of mucus is a defense mechanism that traps pathogens, particles, and toxic chemicals floating in the air. The mucus also assists in the removal of endogenous debris, including dying epithelial cells and leukocytes, which are expelled from the airways by cilia and cough 104. Extracellular gels made of water and mucins (highly glycosylated proteins) are the most important components of mucus. Paradoxically, although the lack of a mucus barrier makes the lungs vulnerable, excess mucus or an impaired clearance thereof is the pathogenesis of many common airway diseases, including PF 104. Excessive mucin secretion or surface fluid volume imbalance may increase the solid concentration by up to 15%, resulting in sticky and elastic mucus that is not easily removed 105. Mucin 5B (*MUC5B*) encodes major gel-forming mucin, which is mainly secreted by the proximal submucosal gland and distal airway, and plays a key role in mucociliary clearance 106. This protein mainly affects the rheological properties of airway mucus and participates in airway defense.

#### Expression of MUC5B in IPF

IPF has long been thought to involve mainly the alveolar region 107. However, abnormal distal airways may also contribute to IPF pathogenesis. In addition to consistent abnormal physiological changes in the distal airways, they have been implicated in ILD 108. A recent histological study showed increased bronchiolar and peribronchiolar inflammation, fibrosis, and thickened bronchial walls in patients with IPF compared with controls 109. The ERBB-YAP axis is considered a driver of distal airway epithelial cell obstruction. This signaling pathway dynamically interacts with MUC5B to increase the degree of distal airway obstruction and is specific to IPF. Distal airway epithelial dysfunction is sufficient to drive primary lung fibroblast activation 108. Visually, the characteristic changes of fibrotic lesions can be seen as subpleural honeycomb-like cysts and predominantly reticular infiltrations in the lower region, as well as a microscopic honeycomb, which represents the airway expanded by the traction of the fibrotic process. They are filled with MUC5B protein and chronic inflammatory cells 110.

*MUC5B* is co-expressed in respiratory bronchiolar airway epithelia 111 and type 2 alveolar epithelial cells 112. In mice, the MUC5B concentration in bronchoalveolar epithelial cells is directly related to the degree and persistence of bleomycin-induced PF and mortality 113. In normal mouse airways that resemble the distal human airway, MUC5B is generated in surface secretory cells in the airways 114. This suggests that it mediates baseline barrier and clearance functions in mice, and that it might have the same role in the human distal airways 115. *MUC5B* expression was increased in tissue sections of distal airways from subjects with PF 116. These data suggest that the distal airways are involved in the pathogenesis of IPF.

#### MUC5B promoter single nucleotide polymorphism and IPF

The gain-of-function promoter variant, rs35705950, is 3 kb upstream of the *MUC5B* transcriptional start site and is the strongest risk factor for the development of f-IPF 117. The conversion from G to T in the 5ʹ region interferes with the binding site of DNA enzymes to transcription factors and upregulates the expression of *MUC5B* 118. The downstream 32 bp of rs35705950 is a highly conserved forehead box A2 (*FOXA2*) binding motif, which is differentially methylated in IPF 119, resulting in increased *MUC5B* expression and rs35705950 being considered a risk allele. This hypermethylation may lead to increased occupancy of *FOXA2* in the binding motif, resulting in increased expression of *MUC5B* 120. The rs35705950 variant enhances *MUC5B* transcription. The resulting post-transcriptional processing can produce a pro-fibrotic response, induce endoplasmic reticulum (ER) stress, and activate the unfolded protein response (UPR) 121. The UPR attempts to restore normal protein folding through three reactions: IRE1/XBP1, PERK/ATF4, and BiP/ATF6 122.

When protein homeostasis is abnormal, UP accumulates in the ER, leading to increased ER pressure, which results in apoptosis. ER stress has been observed in patients with IPF 123. In experimental models of PF, the activation of the UPR in alveolar epithelial cells leads to an epithelial-mesenchymal transition 124 and fibroproliferative pulmonary disease 125. ER stress and apoptosis interfere with normal epithelial responses to injury and repair, which are hallmarks of IPF 126. Whole-genome sequencing analysis has identified an SNP in the promoter region of the *MUC5B* gene, i.e., the rs35705950 variant. It poses a major risk factor for developing IPF, accounting for 30-35% of the risk 127.

Plantier et al. studied the correlation between morphology and transcription factors in the signal transduction system in IPF and found that the uncontrolled expression of *MUC5B* is involved in PF formation 110. Both the homozygous mutation (T/T) and heterozygous mutation (G/T) of the *MUC5B* rs35705950 variant significantly increase the risk of developing f-IPF and s-IPF. By scanning the entire gene sequence of the chromosome 11 p-terminal, 19 SNPs were found to be associated with f-IPF. Finally, rs35705950 located in the promoter region of *MUC5B* was confirmed to be the most strongly associated with familial lung stromal disease and IPF, present in 34% of familial lung stromal disease, 38% of IPF, and only 9% of normal controls, and was considered a risk allele for both diseases 117. The 4-kb promoter region gene of *MUC5B* has three CpG islands and other regulatory sites, suggesting that the expression of *MUC5B* is affected by both genetic and non-genetic factors.

In 2011, two laboratories simultaneously reported that the *MUC5B* promoter variant rs35705950 is the strongest risk factor for the development of f-IPF and s-IPF 128 and appears to be similarly prevalent in patients with f-IPF and s-IPF 117 (approximately 50-60% of individuals with FIP or IPF). This factor increases the risk of heterozygosity by six-fold and that of homozygosity by 20-fold, suggesting that both forms of the disease share common genetic variations. IPF is caused by excessive and continuous lung injury or repair abnormalities 122.

In addition, common exposure and basic biological processes can affect *MUC5B* expression (Table 1).

#### Pathological role of MUC5B in IPF

In addition to the association between the SNP of the *MUC5B* promoter and IPF, its protein may play a direct role in the pathogenesis of IPF 117. Persistent bronchiolar epithelial injury and the overproduction of MUC5B by airway progenitor cells lead to honeycomb cysts and IPF 116.

First, excessive expression of *MUC5B* can impair mucosal host defense and leads to excessive lung injury caused by inhaled substances. Over time, a reduced clearance rate may lead to the formation of scar tissue, which replaces normal lung tissue. It also results in persistent fiber proliferation, which in turn develops into IPF 117. Exposure to cigarette smoke increases *MUC5B* expression in the lung through the dysregulation of classical signaling pathways (e.g., TGF-β and Wnt), resulting in a fibroblast-like phenotype in alveolar epithelial cells, as demonstrated in mouse (MLE-12) and rat (RLE-6TN) epithelial cells 148. Additionally, several clinical and epidemiological studies have shown that f-IPF and s-IPF are more common in smokers 148. In IPF model mice infected with H1N1, distal airway stem cells proliferate and express keratin 5-positive (*Krt5+*), ablates the intrabronchial region, and assemble into alveolar structures 149. Persistent notch signaling after H1N1 injury leads to failure of regeneration and formation of cellular cysts. KRT5+ cells are present in the fibrotic stroma of IPF and are closely associated with MUC5B-rich cellular cysts 150.

Alveolar stem cells are mainly derived from the distal airway epithelium 151, and MUC5B and dry/progenitor cells participate in the fiber proliferation response. When stem cells attempt to regenerate damaged bronchioles and alveolar epithelium, *MUC5B* overexpression interferes with the interaction between AT2 cells and the underlying matrix, inhibiting alveolar basement membrane re-epithelization. This may exacerbate the ongoing alveolar collapse and fibrosis of adjacent bronchoalveolar units, resulting in chronic fiber proliferation and regeneration process disorders and cellular cyst formation 151. These structures inhibit normal alveolar homeostasis and promote fibroproliferative activity in persistent foci.

Second, under normal conditions, mucociliary clearance depends on effective ciliary movement, adequate hydration of the fluid layer around the cilia, and complete cough 152. The overexpression and accumulation of MUC5B prevent efficient mucus hydration and ciliary function 111 and cause excessive retention of inhalants. Endogenous inflammatory fragments at the bronchoalveolar junction are associated with changes in the osmotic gradient, water moving out of the periciliary layer and into the airway lumen 153, and a reduction in mucociliary clearance. They may also physically affect cilial function 150, enhance inhalation retention. Finally, when sticky protein expression relies on the cystic fibrosis transmembrane conductance regulator anion secretion separation, abnormally thick mucus may be beyond the influence of airway dehydration, which can lead to abnormal host defense 154. The common defects of mucous membranes of inhaled particles may exaggerate the host defense. Facilitate recurrent injury/repair/regeneration mechanism disruption 106, and lead to microscopic scarring and progressive fibrous proliferation in the lungs, lung structural destruction, and IPF development over time 3.

Third, IPF is characterized by the co-overexpression of *MUC5B* and cilium-associated genes 155, which are expressed and operate in ciliated airway epithelial cells of the distal airways and are related to microscopic cellular structures. Matrix metalloproteinase 7 (*MMP7*) is a WNT/β-catenin target gene encoding for a metalloproteinase, which is overexpressed in IPF proliferative epithelial cells. Based on the discovery that MMP7-deficient mice are protected from bleomycin-induced PF, it is speculated that *MMP7* plays a role in promoting fibrosis 156. Plasma *MMP7* concentration in IPF was correlated with two single nucleotide polymorphisms in gene promoter regions, suggesting a potential genetic basis for *MMP7* upregulation.

A known biomarker of IPF can indicate the presence, severity, and prognosis of the disease 157. Plasma *MMP7* has been proposed as a univariate predictor of early interstitial lung disease in first-degree relatives of patients with familial interstitial pneumonia 158. Ciliary *MUC5B* gene expression is associated with *MMP7* concentration and attenuation of ciliary cell differentiation in the airways 159. However, *MUC5B* promoter mutations cause mucus accumulation in the distal region of the lung and impair mucociliary function. Abnormal repair processes after distal airway injury and failure of terminal airway regeneration are activated by cellular cysts.

**Fig 3:**
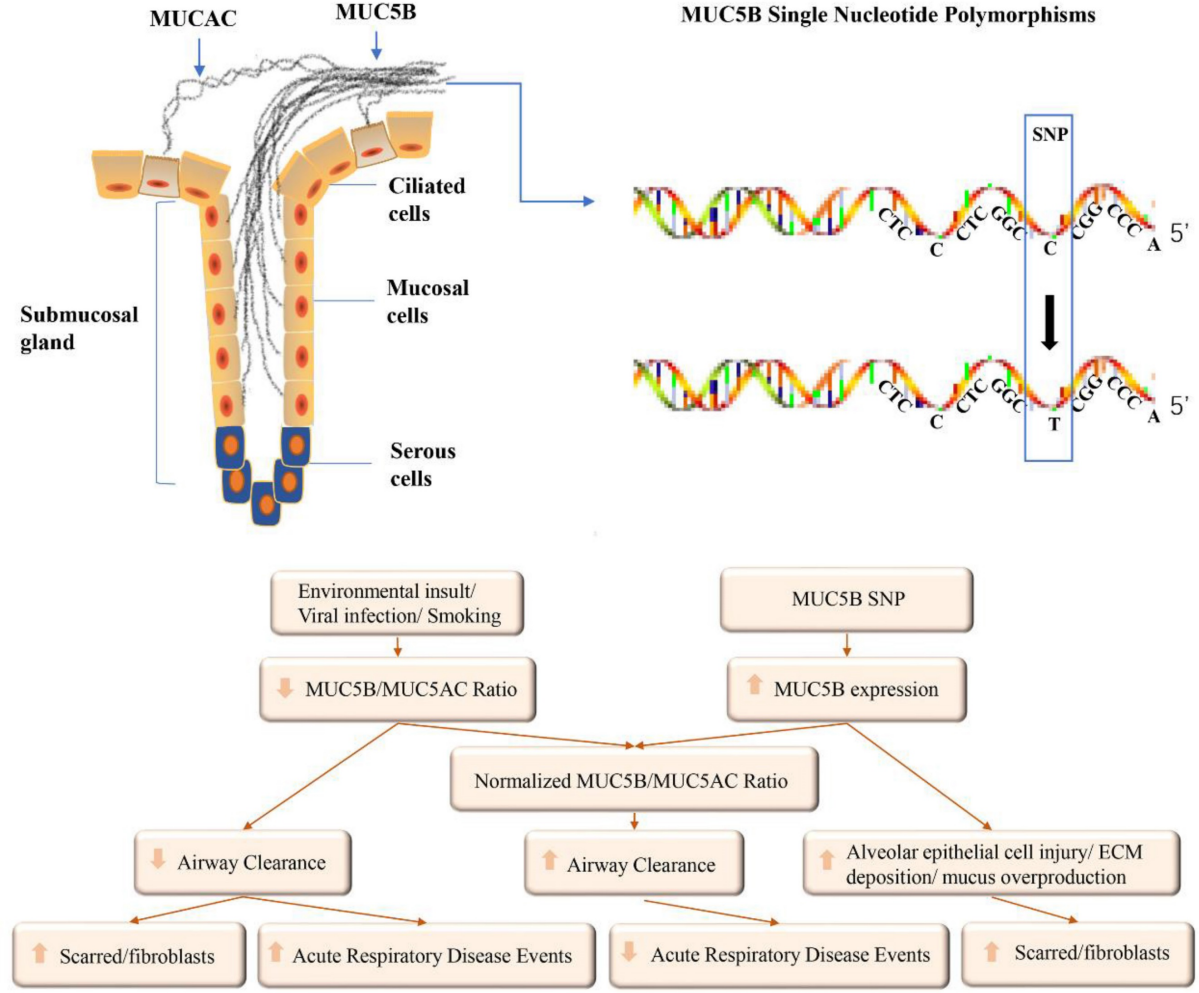
Possible influencing factors of MUC5B expression and its role in airways.

All three mechanisms are reasonable and can act alone or together to cause IIP associated with the *MUC5B* promoter SNP (Fig. 3).

*MUC5B* is mainly produced by submucosal glands and mucous cells on the surface of the airway. SNP were found in the promoter region of *MUC5B*. The abnormal expression of *MUC5B* can be induced by environmental exposure and *MUC5B* SNP. The mucus in the airways can be adjusted by the ability to clear, eventually leading to acute events or pulmonary fibrosis.

*MUC5B* and *TOLLIP* reside at the same genetic locus. If IPF is also associated with the *TOLLIP* promoter SNP, the risk of death is higher 160. The independence of the association signals at 11p15.5 suggests that multiple variants of this locus may be influencing disease susceptibility and course 127. However, this does not prove a relationship between the two genes.

In a meta-analysis of 28 studies, the overall IIP and FIP risk of the TT genotype and the T allele of rs35705950 was significantly increased across all genetic models. These results suggest that *MUC5B* rs35705950 may be a predictor for IIP and FIP susceptibility 161. Preclinical PF (PrePF) is frequently reported in smokers and patients with f-IPF 162, and the polymorphism of the *MUC5B* promoter can be used as an indicator of IPF susceptibility for identifying individuals with PrePF 163 and predicting the radiological progression of PrePF, as well as the prognosis of IPF 164. Participants affected by the IPF variant rs35705950 showed better survival than those who were not 26.

For early asymptomatic f-IPF, the increased MUC5B levels can appear years before the symptoms of PF, and it can be detected in patients with mild PF 165; the variant can serve as an effective new molecular target for intervention during the early stages of IPF. The *MUC5B* promoter variant rs35705950 is predictive of PrePF; however, rs35705950 is present in approximately 19% of the population 117, and IPF rarely occurs (& LT, 0.1%) 166. Therefore, the observed differential expression of *MUC5B* in cases and controls cannot be fully explained 117, and additional biomarkers are needed to identify individuals with PrePF in high-risk populations.

### Surfactant-related genes

Dysfunction and repair of alveolar epithelial cells are considered important components of the pathogenesis of IPF and childhood ILD 167. Nogee et al. first reported a case of nonspecific interstitial pneumonia with exon 4 and amino acid deletion at position 37, which was caused by mutations in the carboxy-terminal region of the gene encoding the surfactant protein of alveolar epithelial cells; the mother of the patient had desquamative interstitial pneumonia 168. F-IPF may also be present in children, accounting for a larger proportion of children with ILD 169.

**Fig 4:**
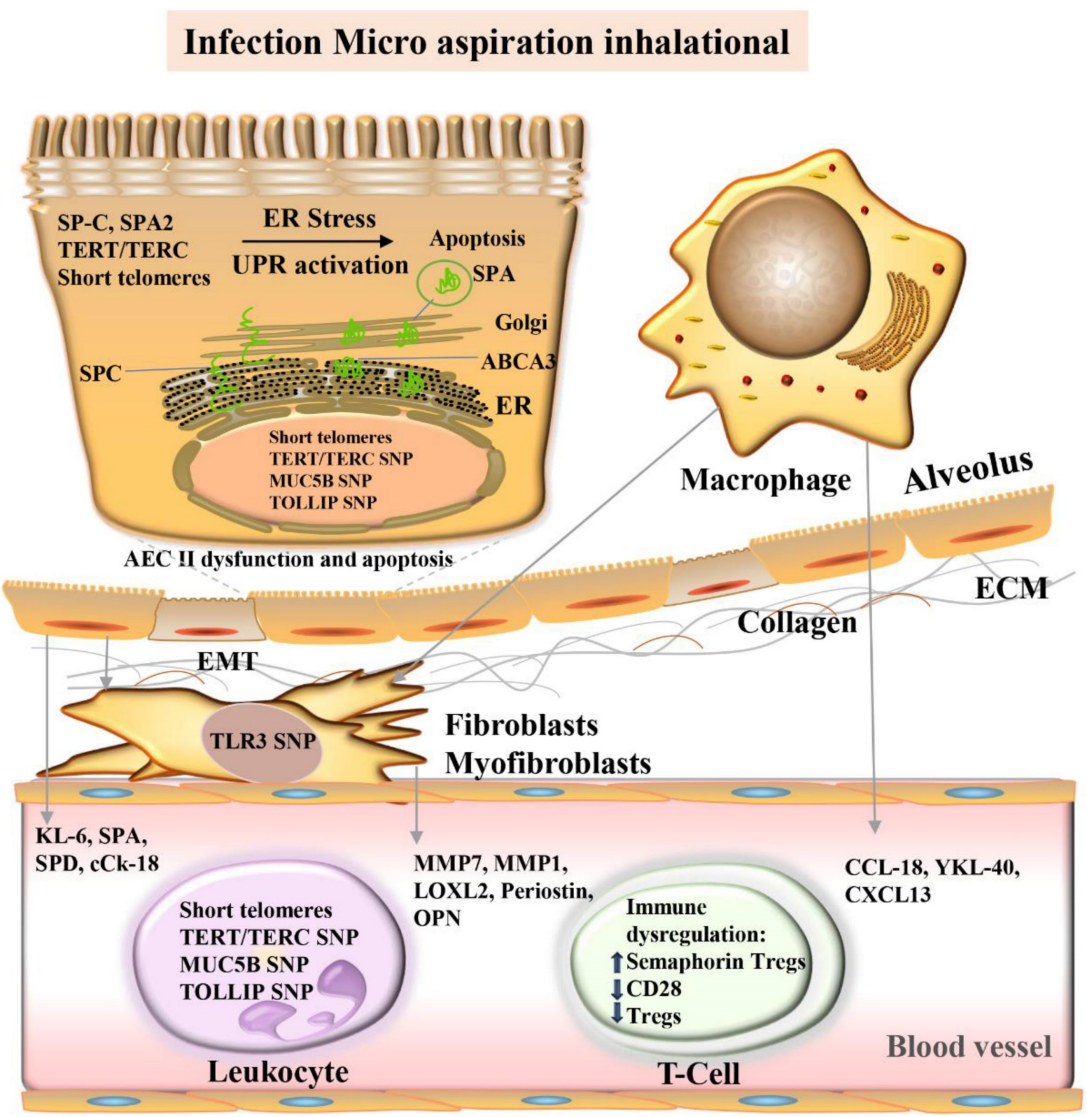
Core mechanisms and candidate molecular biomarkers of idiopathic pulmonary fibrosis.

The central role of AT2 cells in IPF is to generate pulmonary surfactant 170, and AT2 cells self-renew as progenitor cells and transdifferentiate into AT1 cells to maintain alveolar stability 171. Rare mutations in the components of the surfactant system, including surfactant-associated protein C (SP-C), SP-A, and ABCA3 lipid transporters, provide new clues for IPF caused by AT2 cell dysfunction (Fig. 4). More than 60 rare mutations in the SP-C gene (*SFTPC*) have been identified in affected children and adults combined 168, two mutations in the SP-A2 gene (*SFTPA2*), although present in less than 5% of s-IPF, and 150 mutations in *ABCA3* 172. Protein aggregation, ER stress, proinflammatory/profibrotic cytokine processing, altered macroautophagy, and apoptosis occur in model systems with *SFTPC*, *SFTPA*, and *ABCA3* mutations. In humans, they occur in lung and AT2 epithelial cells of patients with f-IPF and s-IPF alike 173.

Pictured are the alveolar lumen (top), epithelial cell layer (tan), interstitial space (white), and capillaries (bottom, pink). Damage to alveolar epithelial cells by multiple stimuli (blue boxes) leads to epithelial dysfunction, fibrosis/extracellular matrix (ECM) deposition, and immune dysregulation. Proposed molecular biomarkers for these processes include blood proteins, genomic markers, and blood cells (see light purple/light green boxes). *ABCA3*, ATP-binding cassette class A3 lipid transporter; AEC, Alveolar epithelial cells; cCK18, cleaved cytokeratin 18; CCL-18, CC chemokine ligand-18; CXCL, chemokine ligand; ECM, extracellular matrix; EMT, epithelial-mesenchymal transition; ER, endoplasmic reticulum; MMP, Matrix metalloproteinase; KL-6, Krebs von den Lungen-6; *LOXL2*, Lysyl oxidase-like 2; *MUC5B*, mucin 5B; SNP Single Nucleotide Polymorphism; OPN, osteopontin; SPA, surfactant protein A; SPA2, surfactant protein A2; SPC, surfactant protein C; SPD, surfactant protein D; TERT telomere RNA component; TERC, telomere reverse transcriptase; *TLR3*, Toll-like receptor 3; TOLLIP, Toll-interacting protein; Treg, regulatory T cells; UPR, unfolded protein response.

Rare mutations of surfactant system components (*SP-C*, *SP-A*, and *ABCA3*) cause stress and activate the unfolded protein response, AT2 dysfunction, and apoptosis, thereby promoting epithelial-mesenchymal transformation and the development of PF. ER, endoplasmic reticulum; TERT, telomere reverse transcriptase; TERC, telomere RNA component; MUC5B, Mucin 5B; SNP, single nucleotide polymorphism; TOLLIP, toll-interacting protein.

#### SP-C

SP-C is a highly hydrophobic protein present in lung surfactants and is produced by ER integration of membrane protein precursors in AT2. The gene encoding *SFTPC* is located on chromosome 8P and organized into six exons (encoded from I to V, with VI untranslated) and five introns, which produce an mRNA that encodes a 21-kDa proprotein (pro-SP-C 21) at either 191 or 197 amino acids. The pro-SP-C 21 COOH terminal, known as the BRICHOS domain (residue 90-197), is the carboxy-terminal region of the pro-SP-C proprotein. Most *SFTPC* mutations are located in the intrapeptide disulfide bond within the pro-SP-C COOH terminal (BRICHOS) domain 174. *SFTPC* BRICHOS mutants are retained by the ER, inhibit the proteasome, and activate the UPR 175, a set of protective biochemical pathways aimed at matching the protein capacity of the ER. The resulting cytokine processing, apoptosis, and functional disruption of progenitor cells lead to PF under AT2 and AEC lumen ER stress 176.

Although *SFTPC* mutations occur in less than 5% of patients with sporadic IPF, these rare mutants enhance our understanding of AT2 cell and AEC cavity biology in PF. Lawson et al. demonstrated evidence of UPR activation markers (including heavy-chain- binding protein (BiP), ER degradation enhancing α-mannosidase-like proteins (EDEM), and XBP1) in patients with f-IPF expressing the L188Q *SFTPC* mutation and samples from non-SFTPC f-IPF and s-IPF cases 177. Korfei et al. extended these findings by reporting molecular signatures of multiple UPR pathways and apoptosis in AT2 cells from patients with s-IPF and UIP pathologic features 178. These included activating transcription factor 6 (ATF6), ATF4, XBP1, C/EBP-homologous protein (CHOP), and caspase 3.

Chronic ER stress in the alveolar epithelium may represent a broad mechanism of ILD pathogenesis 125. In a transgenic mouse model, the diphtheria toxin receptor was expressed exclusively in AT2 cells, using the SFTPC promoter. The administration of the toxin induced AT2 cell death and increased lung collagen deposition, leading to spontaneous PF 179.

The missense substitution G.1286T>C, which replaces isoleucine at position 73 in the SP-C preprotein (pre-SP-C I73T) with threonine, is the most common SFTPC mutation associated with ILD 180. The deposition of pre-SP-C I73T in the plasma membrane and subsequent accumulation of misprocessed allotypes result in a dysfunctional cellular phenotype characterized by late macrophage arrest, impaired mitophagy, and defective cellular protein homeostasis 181. In the bleomycin-induced mouse model of PF, immunohistochemistry of pre-SP-C I73T confirmed that the proliferative cells in the fibrotic and tissue deformable areas are type II AEC, similar to human UIP, and that the fibrotic areas in the AEC cavity are positive for the expression of pre-SP-C 182.

Furthermore, the expression of mutant SFTPC alveolar epithelial cells of mice drives the outbreak of a spontaneous FP. This demonstrates that AT2 cells can promote the creation of characteristic endophenotypes of IPF/UIP, including abnormal tissue remodeling to collagen deposition, AT2 cell proliferation, α-smooth muscle actin-positive cells, and limiting lung physiology 183. SP-C null mice are motile at birth and grow normally in the absence of a pulmonary phenotype 184; however, they show a higher sensitivity to bleomycin-induced fibrosis 185 and respiratory syncytial virus infection 186. When the original SP-C-null-mouse strain was backcrossed to a different genetic background, the resulting loss of SP-C was associated with spontaneous inflammation and lung remodeling 187, suggesting that SP-C deficiency is a disease regulator.

#### SP-A

SP-A, the most abundant surfactant-associated protein, is an intra-luminal multifunctional sialic acid glycoprotein. It has a molecular mass of 28-36 kDa and contains a COOH-terminal C-type lectin motif, triple helix collagen domain, and carbohydrate recognition domain 188. Mutations in *SFTPA*, encoding one of the two isomers of surfactant proteins, are associated with the heritability of f-IPF (mutations *SFTPA1* and *SFTPA2*, 4.5 kb long, each, and located on chromosome 10) 189. *In vivo*, the expression of disease-associated SFTPA1 mutations leads to AT2 UPR activation, necrotizing apoptosis, and IPF, which are associated with ER stress-induced c-Jun N-terminal kinase (JNK) signaling 190.

Takezaki et al. detected a homozygous mutation of the *SFTPA1* locus in two Japanese patients with f-IPF 190. These patients exhibited a significant decrease in downstream SP-A secretion and an increased susceptibility to the influenza virus, which accelerated the progression of IPF. The overexpression of receptor interacting serine/threonine kinase 3 (*RIPK3*) and mixed lineage kinase-like (*MLKL*) was detected in AT2 of *SFTPA1* homozygous knockout mice, but both *RIPK3* and *MLKL* could significantly improve the progression of IPF, suggesting that the *SFTPA1* homozygous mutant reduced* SP-A* production.

Furthermore, we found that the loss of *SFTPA1* leads to ER stress, which results in the overexpression of inositol requiring enzyme 1α (*IRE1α*) and increases *JNK* phosphorylation, thereby upregulating *RIPK3* expression. The inhibition of *IRE1α* and *JNK* significantly inhibited *RIPK3* expression and prevented the progression of PF. In contrast, these processes can be reversed by *RIPK3* overexpression; this finding supports the idea that family genetic history can increase the susceptibility of IPF to external stimuli, aggravate the damage caused by the fibrotic process of AT2, and mediate the aggravation of IPF. Thus, *RIPK3* may be an important target to treat IPF.

#### ABCA3

Clinical mutations in *ABCA3*, encoding for an ATP-dependent transporter that converts phosphatidylcholine and cholesterol into lysosome-associated organelles and is critical for lamellar formation, link lamellar body dysfunction to fibrotic lung disease. Human *ABCA3* has been mapped to chromosome 16p13.3 and encodes a protein of 1,704 amino acids 191. Although *ABCA3* mRNA has been detected in many tissues, its mRNA is highly expressed in AT2 cells 192. Homozygous *ABCA3* mutations lead to lamellar body deletion and neonatal respiratory failure in both humans and mice 193, indicating its importance in the production of pulmonary surfactants. Heterozygous *ABCA3* mutations exist with partial loss of function owing to ER retention (type I), improper functioning of the lipid pump (type II), or both (type I/II compound heterozygotes). These mutants act as genetic modifiers of pulmonary disease associated with *SFTPC* mutations in children 194.

The heterozygous variants, I73T *SFTPC* and D123N *ABCA*, increase disease penetrance, suggesting an interaction between these genotypes; however, the molecular mechanism between them remains unclear 195. In addition to the loss of function, several clinical *ABCA3* mutations are associated with PF, and cell phenotypes derived from *ABCA3* mutations are found in isotypes, ER retention, and misallocation homotypes based on protein behavior 167. However, the mechanistic links among *ABCA3* mutants, changes in cell quality control, and fibrosis require further investigation.

## Conclusions

F-IPF is rarely identified in patients before disease progression, which limits our ability to study mechanisms. Identifying diagnostic and prognostic biomarkers and mechanisms helps to understand the pathogenesis of early f-IPF. Recent studies have increased the understanding of the underlying genetic susceptibility to PF and the pathogenesis of this disease. Genomic factors can also influence the development of f-IPF and the observed heterogeneity in pathogenicity. Genetically susceptible broncho-alveoli may sustain disproportionate damage at different time points owing to different environmental exposures and can eventually manifest as PF.

A positive gene diagnosis may be significant for the early detection, prognosis, and risk assessment of close relatives, and genetic testing may be beneficial for patients with high FIP and positive gene probability 101. If these changes predict disease progression, they will provide the strongest evidence to date that there is a long but identifiable pre-symptomatic period during which targeted therapies can prevent the progression of PF. There are no standardized guidelines for the timing of genetic tests for patients with IPF 3; however, experts generally agree that individualized genetic testing should be performed case by case.

The relationship between the mechanisms of rare and common genetic variations in FIP is not fully understood; they are thought to act synergistically to modulate disease-related phenotypes, such as telomere shortening. In addition, when considering lung transplantation, a positive genetic diagnosis in patients with f-IPF helps predict disease course and assess the risks. Further research should be aimed at revealing the underlying and pathogenic genetic factors and epigenetic changes in patients with f-IPF to improve the usefulness of precision therapies.

## Figures and Tables

**Table 1:** Regulation of MUC5B expression in airway epithelial cells.

Species	Effector	Pathway	Transcriptional factors	Secretion	References
Bacteria spp	LPS/staphylococcus enterotoxin	MAPKs	CREB/AP1/SP1/ NF-κB	+	(129-137)
Cytokines	IL-1 β		NF-κB	+	(132, 138, 139)
	IL-17A		NF-κB	+	(132, 139, 140)
	IL-6	MAPKs	CREB/AP1/SP1/ NF-κB	+	(131-135, 140, 141)
		STAT3	FOX	Decrease	(119, 142, 143)
	IL-13	STAT6	FOXA2	Decrease	(119, 144, 145)
Lipid Mediator	PGD2	MAPKs	CREB/AP1/SP1/ NF-κB	+	(119, 131-135, 146)
Oxidation factors	ROS	MAPKs	NF-κB	+	(147)

Note: Different stimuli in airway epithelial cells can regulate *MUC5B* expression, including certain bacterial components, some interleukins, oxidative factors, lipid mediators, which may induce *MUC5B* overexpression through MAPK, STAT3, or STAT6 pathways. Some important transcription factors are also directly or indirectly involved in *MUC5B* overexpression.

## Abbreviations

ABCA: adenosine triphosphate binding box subfamily A3
AT2: alveolar type II
ATF: activating transcription factor 6
BRICHO: bond within the pro-SP-C COOH terminal
CHO: C/EBP-homologous protein
DC: dyskeratosis congenita
DDR: DNA damage response
DKC1: dyskeratosis congenita 1
EDEM: ER degradation-enhancing alpha-mannosidase-like proteins
ER: endoplasmic reticulum
f-IPF: familial idiopathic pulmonary fibrosis
ILD: interstitial lung disease
IIP: idiopathic interstitial pneumonia
IRE1α: inositol requiring enzyme 1α
JNK: c-Jun N-terminal kinase
MLKL: mixed lineage kinase-like
MMP7: matrix metalloproteinase 7
MUC5B: mucin 5B
NAF1: nuclear assembly factor 1
PARN: poly(A)-specific ribonuclease
PBMCs: peripheral blood mononuclear cells
PrePF: preclinical PF
RIPK3: receptor interacting serine/threonine kinase 3
RTEL1: regulator of telomere length 1
snoRNAs: small nucleolar RNAs
SNP: single nucleotide polymorphism
SFTPA2: surface-active protein A2
SFTPC: surface-active protein C
s-IPF: sporadic IPF
SP-A: surfactant protein A
SP-C: surfactant-associated protein C
TERC: telomerase RNA component
TERT: telomerase reverse transcriptase
TINF2: telomere repeat binding factor 1-interacting nuclear factor 2
TPP1: tripeptidyl-peptidase 1
TRG: T cell receptor gamma locus
UIP: usual interstitial pneumonia
UPR: unfolded protein response
ZCCHC8: zinc finger CCHC-type containing 8
